# Zinner syndrome: A unique triad of mesonephric duct abnormalities as an unusual cause of urinary symptoms in late adolescence

**DOI:** 10.4103/0970-1591.70592

**Published:** 2010

**Authors:** Nitin P Ghonge, Bharat Aggarwal, Amit Kumar Sahu

**Affiliations:** Body Imaging Division, Diwan Chand Satyapal Aggarwal Imaging Research Center, New Delhi, India

**Keywords:** Ejaculatory duct obstruction, mesonephric duct abnormality, seminal vesicle cyst, Zinner syndrome

## Abstract

The present article reports a triad of right renal agenesis, ipsilateral seminal vesicle cyst, and ejaculatory duct obstruction (Zinner syndrome) in a 19-year boy who presented with urinary symptoms. A detailed review of the relevant literature is also presented.

## INTRODUCTION

Zinner’s syndrome is a triad of mullerian duct abnormality comprising of unilateral renal agenesis, ipsilateral seminal vesicle cyst, and ejaculatory duct obstruction.[1] The patients are usually diagnosed at third or fourth decade of life and often present with infertility.[2,3] The case illustrated in this article is unique in terms of early age of presentation during late adolescence with predominantly urinary symptoms.

## CASE REPORT

**Figure 1:**
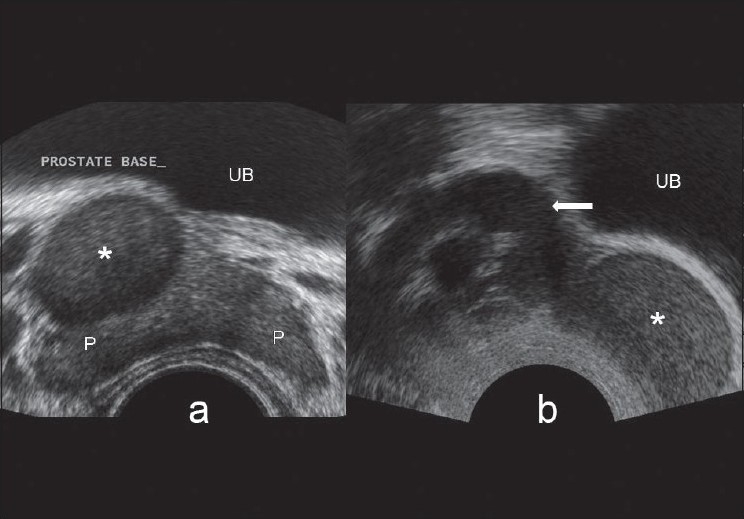
(a) Trans-rectal ultrasound transverse image showing a large cyst (*) with internal echoes in the region of right seminal vesicle. (b) Trans-rectal ultrasound parasagittal image showing a large cyst (*) with internal echoes in the region of right seminal vesicle. Gross dilatation of the proximal part of right seminal vesicle is also seen (arrow). P, prostate; UB, urinary bladder

**Figure 2:**
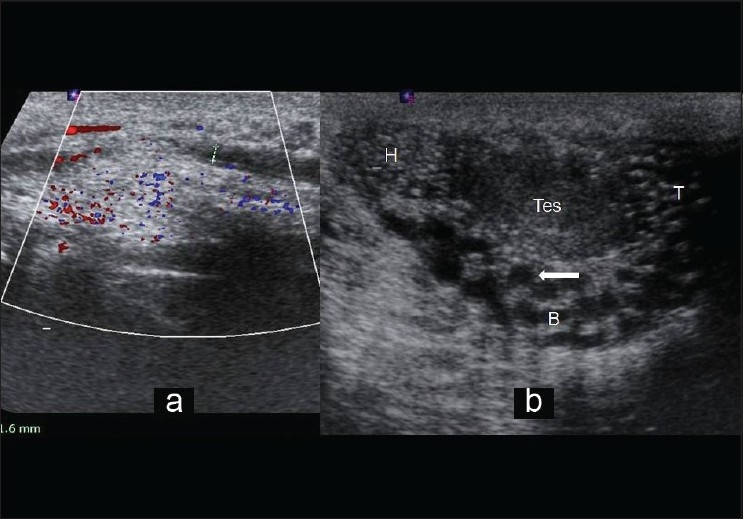
(a) High-frequency ultrasound longitudinal image of right spermatic cord showing dilatation of the right vas deferens in the region of right inguinal canal. (b) High-frequency ultrasound longitudinal image of right scrotal sac showing dilated seminiferous tubules along the complete length of epididymis (arrow). H, head; B, body; T, tail of epididymis; Tes, right testis

**Figure 3:**
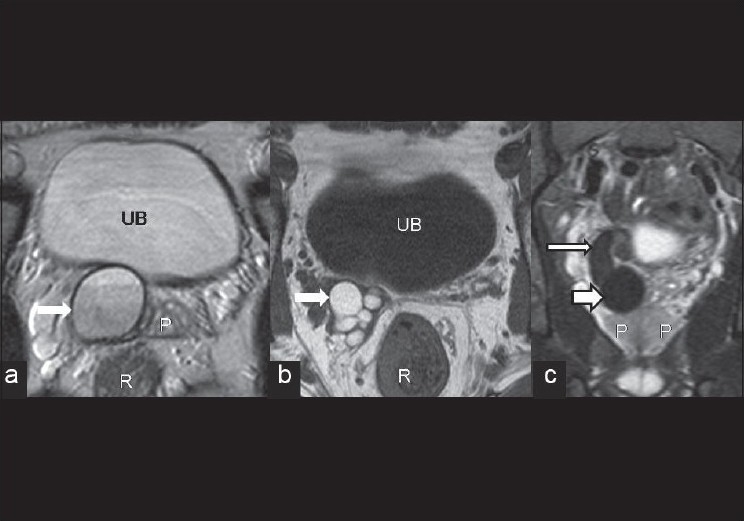
(a) Abdominal MRI T2-weighted axial image acquired using TSE sequence showing a large thick-walled cyst in right seminal vesicle with hyperintense contents and presence of fluid–fluid level within (arrow). (b) Abdominal MRI T1-weighted axial image acquired through a more cranial level showing hyperintense contents in the proximal part of dilated right seminal vesicle (arrow). (c) STIR coronal MRI showing continuity of cyst (short broad arrow) with the right seminal vesicle (long thin arrow). P, prostate; R, rectum; UB, urinary bladder

The illustrated case is a 19-year boy who presented with dysuria, increased frequency of micturition, intermittent pain in scrotum and perineum, and painful ejaculation since 1 year. The patient also had episodes of hematospermia and hematuria. The patient was unmarried and denied any sexual activity. On urine analysis, there were 10–20 isomorphic erythrocytes per high-power field, without any proteinuria or pus cells in any examination. The complete blood cell count, serum biochemistry, and coagulation profile were within normal limits. Semen analysis showed ejaculate volume of less than 1 ml, sperm count of 400,000/ml, alkaline pH, and fructose 1.1 g/l. Hormone analysis showed luteinizing hormone (LH), follicle-stimulating hormone (FSH), and testosterone within normal limits. Trans-abdominal ultrasound showed nonvisualization of right kidney in the right renal fossa or elsewhere in the abdominal cavity and suggested right renal agenesis. The left kidney also showed compensatory hypertrophy. A well-defined rounded anechoic lesion is also detected in right periprostatic region. Further evaluation of anechoic lesion with trans-rectal ultrasound confirmed a large cyst measuring 25 mm in the region of right seminal vesicle [Figure 1]. The normal right seminal vesicle was not identified. There was significant compression over the right ejaculatory duct, which was not identified. The distal part of vas deferens was also compressed. The vas deferens was dilated all along its course in spermatic cord [Figure 2a]. High-frequency ultrasound of inguinoscrotal region also showed significant dilatation of seminiferous tubules in right epididymis involving the head, body, and tail region [Figure 2b]. No definite dilatation of vas deferens or ejaculatory duct or any abnormality in the seminal vesicle was seen on left side. The testes were normal on both sides. MRI of pelvis and inguinoscrotal region was also performed for further evaluation. The right renal agenesis was confirmed (not shown in figures). In addition, the cyst contents showed hyperintense signal on T2-weighted MRI with the presence of fluid–fluid level, which accounts for the presence of intracystic bleed [Figure 3a]. The proximal part of right seminal vesicle was dilated and shows hyperintense signal on T1-weighted images [Figure 3b]. MRI also showed the continuity between the dilated proximal seminal vesicle and the seminal vesicle cyst on the right side [Figure 3c]. In view of the above-mentioned clinical and radiological findings, the diagnosis of Zinner syndrome was made. Trans-urethral resection of ejaculatory duct cyst was advised as a definitive treatment option and close follow-up as an alternative option. The patient preferred the conservative option and is presently being followed-up.

## DISCUSSION

Congenital malformations of seminal vesicle are often associated with ipsilateral upper urinary tract, as both ureteral buds and seminal vesicles originate from the mesonephric (Wolfian) duct.[2,4] The association was first described by Zinner in 1914 and till 2000, about hundred cases had been reported.[1,2,4] Zinner syndrome is also considered to be the male counterpart of Mayer-Rokitansky-Kustner-Hauser (MRKH) syndrome (uterovaginal aplasia) seen in females.[3] Casey *et al*. had reported a unique pentad of mesonephric duct abnormalities on imaging, including cystic dysplasia of rete testis, seminal vesicle cyst, ipsilateral renal agenesis, partial hemitrigonal development, and epididymal dilatation.[5]

Mesonephric (Wolfian) duct is a paired organ found in humans during embryogenesis. In males, it develops into hemitrigone, bladder neck, urethra (proximal to the external sphincter), seminal vesicle, vas deferens, efferent ducts, epididymis, paradidymis, and appendix epididymis under the influence of testosterone and anti-mullerian hormone.[6] An insult during the first trimester adversely affects the embryogenesis of kidney, ureter, seminal vesicle, and vas deferens. Maldevelopment of the distal part of mesonephric duct leads to atresia of the ejaculatory duct (leading to the obstruction and cystic dilatation of seminal vesicle) and abnormal ureteral budding (leading to renal agenesis or dysplasia). The obstruction at the level of ejaculatory duct leads to gradual accumulation of secretions in the seminal vesicle with consequent cyst formation. This sequential developmental pathology attributes to azoo/oligozoo-spermia, which may manifest as primary infertility. Depending on the size of the cysts, there may be pressure effects over the adjoining structures and account for the pelvic and perineal pain. An ultrasound-based study involving 280,000 children in Taipei reported presumed seminal vesicle cysts with approximate incidence of 0.0046% in patients with ipsilateral renal agenesis or dysplasia.[7] Presence or absence of associated renal agenesis or dysplasia depends on the time of insult during the embryogenesis, as prior to 7 weeks of gestation (before the ureteric bud appears) associated renal agenesis is very likely.[8]

Most patients with this group of mesonephric duct anomalies are asymptomatic until the third or fourth decade of life and often manifest during the period of high sexual or reproductive activity. The seminal vesicle cyst in asymptomatic patients is often less than 5 cm and discovered incidentally during digital rectal examination or during cross-sectional imaging. The patients present with pelvic or perineal pain, dysuria, painful ejaculation, chronic recurrent epididymitis/prostatitis, and occasionally infertility. Some cases have nonspecific symptoms such as prostatism, urinary urgency, dysuria, painful ejaculation, and perineal discomfort.[2,9,10] Cysts larger than 12 mm are termed as giant cysts as they are also likely to cause bladder and colonic obstruction.[11] Rarely, malignant transformation in the seminal vesicle cyst has also been reported.[12]

Patient illustrated in this article, however, presented at an early age of 19 years predominantly with urinary symptoms and associated vague pain in scrotal and perineal regions. The diagnosis was facilitated with trans-abdominal ultrasound, which showed a small cystic lesion along the right superolateral aspect of the prostate, which prompted the trans-rectal ultrasound and MRI studies.

On ultrasound, the seminal vesicle cyst is seen as an anechoic pelvic mass with a thick and irregular wall, which may or may not show mural calcification. Presence of internal echoes suggests prior hemorrhage or infection.[13,14] The cyst may be initially detected on trans-abdominal ultrasound study and may be further evaluated on trans-rectal ultrasound. Excretory urography can show associated ipsilateral renal agenesis or dysgenesis.[13] Seminal vesicle cyst may also be seen as an extrinsic smooth-walled filling defect along the inferolateral bladder surface. The findings on vasovesiculography include dilatation, deformity of seminal vesicle, ejaculatory duct stenosis, and reflux of contrast material in an ipsilateral ectopic ureter. The communication between the components of mesonephric duct can be delineated.[15]

A seminal vesicle cyst can be seen on computed tomography (CT) as a well-defined retrovesicular mass of water or near-water attenuation that is often seen just superior to the prostate gland.[2] Precise delineation of the renal anomalies and the altered pelvic anatomy is feasible with CT. The seminal vesicle cyst shows thick irregular wall or hyperdense contents with enlargement of the ipsilateral seminal vesicle.[16] Owing to multiplanar ability, excellent soft tissue resolution, and use of nonionising radiation, MRI is the ideal imaging study to evaluate malformations of the mesonephric duct.[15,17] MRI appearance of seminal vesicle cyst is similar to a cyst located elsewhere in the body as it appears hypointense on T1-weighted and hyperintense on T2-weighted images. Presence of protein-rich contents or previous episode of intracystic bleed may lead to hyperintense signal on T1-weighted images and hypointense signal on T2-weighted images.[2,13] MRI has been shown to be accurate in differentiating seminal vesicle cysts from other pelvic cystic malformations.[18] Presence of a convoluted tail connecting the cystic abnormality to the seminal vesicle and the presence of fluid of high signal intensity on T1-weighted images favor the seminal vesicular origin of the cystic lesion.

Cysts and cystic dilatation of seminal vesicles can be mimicked by several pelvic lesions and would require accurate differentiation. These include true cysts of prostate gland, prostatic utricle cysts, ejaculatory duct cysts, mullerian duct cysts, hydronephrotic pelvic kidneys, bladder diverticula, and ureteroceles. The differentiation is often based on the position (median, para-median, or lateral), intralesional content, associated findings in urogenital system, and the imaging characteristics. Mullerian duct cysts and ejaculatory duct cysts are midline in location. The presence of spermatozoa in the aspirate may differentiate seminal vesicle cysts from mullerian duct cysts. Diverticulosis of ampulla of vas deferens and ectopic ureterocele are more laterally located. MRI is also helpful for accurate preoperative surgical planning for seminal vesicle cyst excision. Surgical excision of a seminal vesicle cyst depends on the size and location of the cyst and presence of clinical symptoms.

The triad of mullerian duct abnormality comprising of unilateral renal agenesis, ipsilateral seminal vesicle cyst, and ejaculatory duct obstruction (Zinner syndrome), therefore, constitutes an uncommon but important diagnostic consideration in young age when the patient presents with recurrent urinary symptoms. The modern-day imaging techniques have facilitated the early diagnosis of this entity.
